# Spinal Analgesia Versus Intravenous Low-Dose Oxycodone for Pain Management After Robotic Hysterectomy: Preliminary Results from an ERAS Institution

**DOI:** 10.3390/jcm14196957

**Published:** 2025-10-01

**Authors:** Elisa Peano, Roberta Rosso, Katia Audisio, Giuseppe Coletta, Andrea Puppo, Barbara Franzoso

**Affiliations:** 1 Department of Gynecology and Obstetrics, Azienda Sanitaria Ospedaliera S. Croce e Carle, 12100 Cuneo, Italy; peano.el@ospedale.cuneo.it (E.P.); puppo.a@ospedale.cuneo.it (A.P.); 2 Department of Anesthesiology, University of Turin, 10124 Torino, Italy; katia.audi@hotmail.it; 3 Department of Anesthesiology, Azienda Sanitaria Ospedaliera S. Croce e Carle, 12100 Cuneo, Italy; coletta.g@ospedale.cuneo.it (G.C.); franzoso.b@ospedale.cuneo.it (B.F.)

**Keywords:** enhanced recovery after surgery, intraoperative opioids, postoperative pain management, robotic surgery, spinal analgesia

## Abstract

**Background:** Robotic hysterectomy and Enhanced Recovery After Surgery (ERAS) are two significant improvements in gynecologic surgery, both associated with decreased postoperative pain and faster recovery. Spinal analgesia guarantees excellent pain coverage; however, its appropriateness in robotic procedures is still controversial. The aim of the study was to compare postoperative pain control after robotic hysterectomy in patients receiving spinal analgesia versus intravenous low-dose oxycodone. **Methods:** Consecutive patients undergoing robotic hysterectomy from January 2022 to July 2023 were included in the analysis. Until August 2022, patients received spinal analgesia, while from September 2022, low-dose oxycodone was administered intraoperatively. All patients were managed following the ERAS protocol. Primary outcomes were the VAS pain score and opioid rescue use, while secondary outcomes included postoperative nausea and vomiting (PONV), mobilization, oral intake, and length of hospital stay (LOS). **Results:** Of 114 patients, 67 (58.8%) received spinal analgesia and 47 (41.2%) received intravenous low-dose oxycodone. No differences were reported in the VAS pain score at day 0 (1.5 ± 1.6 vs. 1.6 ± 2.2, *p* = 0.78) and day 1 (2.0 ± 2.1 vs. 1.3 ± 1.8, *p* = 0.07). At day 2, the VAS pain score was 1.4 ± 1.6 in the spinal analgesia group and 0.7 ± 1.0 in the oxycodone group (*p* = 0.01). No differences were reported in the need for opioid rescue at days 1 and 2 (*p* = 1.00). At day 0, 26 patients (38.8%) experienced PONV in the spinal analgesia group versus 8 (17.0%) in the oxycodone group (*p* = 0.01). **Conclusions:** Patients receiving intraoperative low-dose oxycodone experienced comparable satisfactory postoperative pain control with a lower incidence of PONV when compared to the spinal analgesia group.

## 1. Introduction

Gynecological surgical techniques have significantly developed during the last decades, evolving from open to minimally invasive approaches, such as laparoscopy and, subsequently, robotic surgery [1]. This transition has profoundly changed the perioperative management of patients, offering many benefits not only in terms of surgical outcomes but also in the overall recovery process. Particularly, robotic surgery combines the advantages of laparoscopy with enhanced visualization and improved ergonomics for the surgeon, leading to more precise dissections, reduced tissue trauma, and less intraoperative blood loss [2,3,4,5,6]. Within this context, perioperative care protocols such as the Enhanced Recovery After Surgery (ERAS) program have gained increasing importance. The ERAS philosophy emphasizes multimodal strategies to minimize surgical stress, reduce postoperative opioid consumption, and accelerate recovery. The combination of the ERAS program with minimally invasive surgery techniques brought notable advantages to patients, including a low complication rate, faster recovery of bowel function, early mobilization, and shorter hospital stays [7,8]. Postoperative pain management remains a cornerstone of successful recovery, also improving the quality of surgical experience from the patient’s perspective [9,10]. Adequate pain control enables patients to earlier initiate oral nutrition and mobilization and consequently facilitates a faster discharge. At the same time, it is increasingly recognized that minimizing opioid consumption in the postoperative period has significant advantages, such as reducing postoperative nausea and vomiting (PONV), hyperalgesia, paralytic ileus, delayed mobilization, and, in rare cases, respiratory depression [11,12]. For all these reasons, the search for analgesic strategies that ensure effective pain relief while minimizing opioid-related side effects is a major topic in current clinical research. Among the analgesic techniques employed in gynecological surgery, spinal analgesia—based on the intrathecal administration of a local anesthetic combined with an opioid, usually morphine—represents a well-established option. It provides excellent pain coverage during the immediate postoperative period and has long been considered a gold standard for open abdominal procedures [13,14,15,16]. Nevertheless, spinal analgesia is not devoid of risks. Potential complications include intraoperative hypotension, headache, urinary retention, pruritus, and, less frequently, spinal hematoma [17,18]. In addition, PONV remains a common side effect and may significantly impair postoperative recovery and patient comfort [19]. In the context of less invasive robotic-assisted procedures, the balance between the benefits and risks of spinal analgesia is still debated. Considering that these procedures are associated with lower surgical trauma and quicker recovery compared to open surgery, the need for invasive analgesic approaches such as spinal analgesia is not fully established. Furthermore, its use can increase operating room turnover time and may not always provide a clear advantage over less invasive alternatives in this specific setting. Furthermore, while the postoperative use of multiple doses of long-acting opioids is not recommended according to the ERAS program, a single low-dose administration of opioids at the beginning of surgery appears to be associated with well-controlled pain in the immediate postoperative period, while minimizing the risks associated with repeated administration [20]. Low-dose oxycodone is associated with favorable pharmacokinetics, an excellent safety profile, and a low incidence of adverse effects. When administered intravenously, oxycodone offers a rapid onset of action within minutes, with a peak analgesic effect occurring within 15–30 min, thus providing effective pain control at the emergence from anesthesia. Moreover, it presents advantageous pharmacokinetic characteristics, as its intravenous administration ensures a rapid onset of analgesia within minutes, with peak effect occurring within 15–30 min, thus providing effective pain control already at the time of emergence from anesthesia [21].

These considerations highlighted the need to reassess the role of spinal analgesia in minimally invasive gynecological surgery and to explore whether less invasive strategies could provide comparable analgesic efficacy with fewer side effects.

The aim of the present study was to compare postoperative pain and incidence of PONV after robotic hysterectomy in patients receiving spinal analgesia versus intravenous low-dose oxycodone after the induction of general anesthesia.

## 2. Materials and Methods

This is a retrospective monocentric study, including consecutive female patients undergoing robotic hysterectomy from January 2022 to July 2023 at the S. Croce e Carle Hospital, Cuneo, Italy. All patients provided informed consent for the use of their data for research purposes. The present study was exempt from Institutional Review Board approval, as it was a retrospective study based on existing data recorded by the investigator in such a way that the identity of the subject cannot be readily ascertained.

Inclusion criteria were age >18 years old, elective surgery, and non-chronic opioid use. All patients were managed according to the ERAS protocol. Until August 2022, pain management was performed with spinal analgesia using morphine 100 mcg and ropivacaine 2.5% 10 mg before induction of general anesthesia. From September 2022, a new clinical pathway was introduced in our Center, and 0.05 mg/kg of oxycodone was administered as a single dose after the induction of general anesthesia.

General anesthesia was induced with remifentanil 0.1–0.25 mcg/kg/min and propofol 2-to-3 mg/kg and maintained with 1.0 minimum alveolar concentration of sevoflurane or propofol 3-to-6 mg/kg/h. Neuromuscular blockade was obtained with rocuronium 0.6-to-1.0 and maintained with a continuous infusion of 0.010-to-0.012 mg/kg/min rocuronium.

Standard monitoring of patients included electrocardiography, pulse oximetry, non-invasive arterial blood pressure, capnography, temperature, EEG-sedline, TOF scan, and urinary output.

All patients received antiemetic prophylaxis with dexamethasone 4 mg, metoclopramide 10 mg, and ondansetron 8 mg intraoperatively. At the end of surgery, paracetamol 1 g and ketorolac 30 mg were administered to all patients, while oral paracetamol 1 gx3/die and oral ibuprofen 600 mgx2/die were set up for the postoperative period. In the postoperative period, different analgesics were used when pain was not controlled by the standard therapy, based on the VAS pain score reported by the patient: for a VAS score > 4 (moderate pain), oral or intravenous paracetamol 1 g was administered; for a VAS score > 6 (severe pain), intravenous ketorolac 30 mg was administered; and for a VAS > 8 (very severe pain), intravenous oxycodone 2 mg was administered. The term “intravenous postoperative opioid rescue use” refers to intravenous oxycodone 2 mg administration. PONV was treated with intravenous metoclopramide 10 mg or intravenous ondansetron 8 mg as a second-line treatment.

The following information was collected for each patient: demographic data, comorbidities, indications for surgery, length of operation, intraoperative blood loss, postoperative mobilization, oral intake, intravenous postoperative opioid rescue use, PONV, and length of stay (LOS). Pain and PONV scores were obtained using a visual analog scale (VAS) ranging from 0 to 10.

Primary outcomes were the VAS pain score at day 0, day 1, and day 2, and intravenous postoperative opioid rescue use, while secondary outcomes included incidence of PONV at day 0, day 1, and day 2, mobilization, oral intake, and LOS. The VAS pain score and the presence of PONV were assessed by the nursing staff every 6 h in the absence of patient complaint and reported on the specific ERAS medical record.

### Statistical Analysis

Continuous variables were expressed as mean and standard deviation (SD) and categorical variables as number and percentage. Differences between groups were assessed using Student's test for continuous variables and chi-square test or Fisher’s exact test for categorical variables. All *p*-values were two-sided, and a *p*-value < 0.05 was considered statistically significant.

## 3. Results

Of 114 patients, 67 (58.8%) received spinal analgesia and 47 (41.2%) received intravenous low-dose oxycodone. Patients in the two groups had comparable baseline and intraoperative characteristics, as reported in Table 1. Mean age at surgery was 63.1 ± 12.8 years in the spinal analgesia group and 62.9 ± 13.1 years in the oxycodone group (*p* = 0.92). Mean body mass index was 27.6 ± 6.9 kg/m^2^ in the spinal analgesia group and 29.0 ± 8.0 kg/m^2^ in the oxycodone group (*p* = 0.35). A total of 58 patients (86.6%) underwent surgery for an oncological condition in the spinal analgesia group versus 45 (95.7%) in the oxycodone group (*p* = 0.10). In the spinal analgesia group, 61 patients (91.0%) received inhalation anesthesia and 6 (9.0%) received total intravenous anesthesia, while in the oxycodone group, all patients received inhalation anesthesia (*p* = 0.04). The mean length of operation was 119.5 ± 35.7 min in the spinal analgesia group and 119.5 ± 38.9 min in the oxycodone group (*p* = 1.00). Intraoperative blood loss was 67.3 ± 25.1 mL in the spinal analgesia group and 77.7 ± 53.0 mL in the oxycodone group (*p* = 0.16).

No differences were reported in the VAS pain score at day 0 (1.5 ± 1.6 vs. 1.6 ± 2.2, *p* = 0.78) and day 1 (2.0 ± 2.1 vs. 1.3 ± 1.8, *p* = 0.07) comparing the spinal analgesia group and the oxycodone group, respectively. At day 2, the VAS pain score was 1.4 ± 1.6 in the spinal analgesia group and 0.7 ± 1.0 in the oxycodone group (*p* = 0.01). No patient needed intravenous opioid rescue in both groups at day 0, day 1, and day 2 (*p* = 1.00).

At day 0, 26 patients (38.8%) experienced PONV in the spinal analgesia group versus 8 (17.0%) in the oxycodone group (*p* = 0.01). At day 1, PONV was found in 12 patients (17.9%) in the spinal analgesia group and 4 patients (8.5%) in the oxycodone group (*p* = 0.18). At day 2, six patients (9.0%) and no patients experienced PONV in the spinal analgesia group and in the oxycodone group, respectively (*p* = 0.04). Solid food at day 0 was tolerated by 30 patients (44.8%) in the spinal analgesia group and 30 patients (63.8%) in the oxycodone group (*p* = 0.04). No differences were found in early mobilization at day 0; it was achieved by 32 patients (47.8%) in the spinal anesthesia group and 17 patients (36.2%) in the oxycodone group (*p* = 0.22). LOS was 2.3 ± 0.5 days in the spinal analgesia group versus 2.3 ± 0.6 days in the oxycodone group (*p* = 1.00). No patients experienced intraoperative or postoperative complications in both groups. The results are summarized in Table 2.

## 4. Discussion

ERAS patients who underwent robotic hysterectomy receiving intravenous low-dose oxycodone at the induction of general anesthesia experienced comparable satisfactory postoperative pain control with a lower incidence of PONV when compared to the spinal analgesia group.

Historically, the gold standard for pain management after open abdominal hysterectomy was epidural analgesia [22,23]. The introduction of laparoscopic surgery, with smaller incisions and less tissue damage, has called into question whether epidural analgesia is still necessary. In fact, epidural analgesia has shown a lower benefit/risk ratio when associated with laparoscopic surgery with significant side effects such as hypotension and increased IV fluid requirements, reduced mobilization, urinary retention, slower return of bowel function, and prolonged LOS [24,25]. Thus, spinal analgesia became the gold standard to control postoperative pain after laparoscopy. A recent cohort study determined that spinal analgesia with 0.8 mL of 0.75% bupivacaine and 200 mcg of morphine prior to laparoscopic hysterectomy significantly reduced postoperative pain and resulted in less opioid consumption [16].

The expansion of robotic surgery brought further advantages for patients and raised further questions about the proper pain management. Robotic systems have wristed instruments which guarantee an enhanced range of motion and a more precise approach to the surgical site, greater accuracy and stability, and improved tissue handling. Furthermore, a three-dimensional camera technology increases the surgeon’s spatial awareness, and a tremor filter avoids involuntary movements [26]. These factors suggest that robotic surgery may be less traumatic and subsequently require a lighter protocol for pain control. Mangalath and colleagues demonstrated that postoperative analgesic requirements, intensive care unit LOS, and time to ambulation were significantly lower in patients undergoing robotic versus laparoscopic abdominal surgery [27].

To date, no standardized protocol for pain management after robotic hysterectomy is recommended, and several institutions have published their own experience. A recent Brazilian study proved that combined intravenous infusion of lignocaine and dexmedetomidine significantly decreased postoperative pain, fentanyl consumption, and improved quality of recovery after robotic hysterectomy [28]. Torup and colleagues compared TAP block with ropivacaine 0.5% 40 mL versus isotonic saline and found no differences in morphine consumption and VAS pain scores after robotic hysterectomy [29].

The present study was designed to test the hypothesis of whether spinal analgesia could be considered an overtreatment for pain management after robotic hysterectomy. As an alternative for spinal analgesia, we investigated the use of intravenous low-dose oxycodone at the induction of general anesthesia, based on our experience and the available literature. While the use of multiple doses of long-acting opioids is not recommended, a single low-dose administration of opioids at the beginning of surgery appears to be associated with well-controlled postoperative pain [20,30,31,32].

In our population, no difference emerged in postoperative VAS pain score between patients receiving spinal analgesia and low-dose intravenous oxycodone, while patients who received spinal analgesia experienced significantly higher rates of PONV. No patient needed opioid rescue, and LOS was comparable in the two groups.

Intraperitoneal pressure, length of surgery, and blood loss are important determinants of postoperative pain, as reported in the literature [33]. The use of low-pressure pneumoperitoneum and the aspiration of residual gas at the end of the procedure have been shown to reduce postoperative abdominal pain, shoulder pain, and analgesic requirements [34]. A randomized controlled trial by Yasir and colleagues comparing low (8 mmHg) and high peritoneal pressure (14 mmHg) in laparoscopic surgery found reduced postoperative pain, lower analgesic requirement, and shorter hospital stay in the first group [35]. In our practice, the mean length of the operation was less than 120 min, very little intraoperative blood loss was reported, and peritoneal pressure was low (10 mmHg). These elements probably improved surgical field healing, thus providing a low VAS pain score in both groups.

The study presents some limitations. Because of the retrospective and non-randomized, time-based allocation design, there is a potential risk of selection bias and unmeasured confounding, which cannot be entirely excluded despite the overall similarity of baseline characteristics between groups. Moreover, the relatively small cohort size may limit the possibility of detecting minor differences or rare complications, and the single-center design of the study may reduce the external validity of the findings. Despite these limitations, the two groups of patients had comparable characteristics at baseline, and the study results are reflective of a real-world patient population. All patients received the same postoperative pain management protocol and were treated by the same anesthesiologist, avoiding individual preferences and ensuring uniformity in pharmacological prescriptions. All surgical procedures were performed by the same team of expert surgeons with a standardized technique, thus minimizing bias due to different surgeons’ experience or lack of uniformity in the procedure. Patients included in the study were all opioid-naive women, thus avoiding the potential interference of prior use of opioids as a confounding factor.

## 5. Conclusions

Our findings suggest that both spinal analgesia and intravenous low-dose oxycodone represent effective strategies for managing postoperative pain after robotic hysterectomy, with the latter associated with a lower incidence of PONV. These preliminary results support the evidence that minimally invasive approaches, when integrated with the ERAS protocol, may reduce the overall analgesic requirement and particularly the need for spinal analgesia, thus minimizing the risks and side effects associated with the procedure. In clinical scenarios where both approaches are viable, non-invasive modalities should be considered the preferred strategy due to their favorable risk/benefit profile. Nevertheless, future research should focus on tailoring approaches to specific clinical situations, as several patient-related and surgical factors may affect postoperative pain perception and influence the balance between risks and benefits of different analgesic modalities. Prospective randomized controlled trials are needed to confirm these preliminary findings, particularly in complex scenarios such as oncological patients, obese women, large uteri, or extensive adhesions, which may require technically challenging procedures, making the choice of analgesia particularly critical.

## Figures and Tables

**Table 1:** Baseline and clinical characteristics.

	Spinal Analgesia (*n* 67)	IV Opioid (*n* 47)	*p* Value
Age, mean ± SD, y	63.1 ± 12.8	62.9 ± 13.1	0.92
BMI, mean ± SD	27.6 ± 6.9	29.0 ± 8.0	0.35
Smoking, *n* (%)	12 (17.9)	6 (12.8)	0.46
Diabetes Mellitus, n (%)	5 (7.5)	6 (12.8)	0.36
Previous MI, *n* (%)	0 (0.0)	0 (0.0)	1.00
Previous CVA, *n* (%)	0 (0.0)	0 (0.0)	1.00
Diagnosis			
Malignant, *n* (%)	58 (86.6)	45 (95.7)	0.10
- Uterine cancer, *n* (%)	44 (65.7)	33 (70.2)	0.61
- Cervical cancer, *n* (%)	9 (13.4)	3 (6.4)	0.35
- Ovarian cancer, *n* (%)	0 (0.0)	2 (4.3)	0.18
- CIN3/HSIL, *n* (%)	4 (6.0)	2 (4.3)	1.00
- Endometrial hyperplasia, *n* (%)	1 (1.5)	5 (10.6)	0.08
Benign, n (%)	9 (13.4)	2 (4.3)	0.10
- Endometriosis, *n* (%)	3 (4.5)	0 (0.0)	0.27
- Adenomyosis, *n* (%)	1 (1.5)	0 (0.0)	1.00
- Leiomyoma, *n* (%)	5 (7.5)	2 (4.3)	0.70
ASA score			
ASA 1	10 (14.9)	1 (2.1)	0.02
ASA 2	45 (67.2)	35 (74.5)	0.40
ASA 3	12 (17.9)	11 (23.4)	0.47
ASA 4	0 (0.0)	0 (0.0)	1.00
General anesthesia			
- Inhalation anesthesia, *n* (%)	61 (91.0)	47 (100.0)	0.04
- TIVA, *n* (%)	6 (9.0)	0 (0.0)	0.04
Length of operation, mean ± SD, min	108.4 ± 33.6	109.1 ± 34.7	1.00
Intraoperative blood loss, mean ± SD, mL	67.3 ± 25.1	77.7 ± 53.0	0.16
Total IV fluids—day 0, mean ± SD, mL	1289.0 ± 294.9	1302.3 ± 353.8	0.87

Note: A total of 23 patients (14 [20.9%] in the spinal analgesia group and 9 [19.1%] in the IV opioid group, *p* value 0.81) did not have a diagnosis of malignancy. BMI: body mass index, CIN: cervical intraepithelial neoplasia, CVA: cerebrovascular accident, HSIL: high-grade squamous intraepithelial lesion, IV: intravenous, MI: myocardial infarction, SD: standard deviation, and TIVA: total intravenous anesthesia.

**Table 2:** Postoperative characteristics.

	Spinal Analgesia (*n* 67)	IV Opioid (*n* 47)	*p* Value
Mobilization—day 0, *n* (%)	32 (47.8)	17 (36.2)	0.22
Mobilization—day 1, mean ± SD, h	4.7 ± 2.3	4.3 ± 2.1	0.35
Mobilization—day 2, mean ± SD, h	4.5 ± 1.9	4.1 ± 2.0	0.28
Tolerating solid food—day 0, *n* (%)	30 (44.8)	30 (63.8)	0.04
Recovery of ADL ability—day 1, *n* (%)	58 (86.6)	32 (68.1)	0.02
VAS pain score—day 0, mean ± SD	1.5 ± 1.6	1.6 ± 2.2	0.78
VAS pain score—day 1, mean ± SD	2.0 ± 2.1	1.3 ± 1.8	0.07
VAS pain score—day 2, mean ± SD	1.4 ± 1.6	0.7 ± 1.0	0.01
Pain control with oral analgesics—day 0, *n* (%)	1	1	1.00
Pain control with oral analgesics—day 1, *n* (%)	56 (83.6)	45 (95.7)	0.07
Pain control with oral analgesics—day 2, *n* (%)	10 (14.9)	2 (4.3)	0.12
IV opioid rescue use—day 0, *n* (%)	1	0	1.00
IV opioid rescue—day 1, *n* (%)	1 (1.5)	1 (2.1)	1.00
IV opioid rescue—day 2, *n* (%)	1 (1.5)	1 (2.1)	1.00
PONV—day 0, *n* (%)	26 (38.8)	8 (17.0)	0.01
- Nausea not requiring treatment, *n* (%)	17 (25.4)	6 (12.8)	0.10
- Nausea requiring treatment, *n* (%)	6 (9.0)	0 (0.0)	0.04
- Vomiting, *n* (%)	3 (4.5)	2 (4.3)	1.00
PONV—day 1, *n* (%)	12 (17.9)	4 8.5)	0.18
- Nausea not requiring treatment, *n* (%)	8 (11.9)	3 (6.4)	0.52
- Nausea requiring treatment, *n* (%)	4 (6.0)	1 (2.1)	0.65
- Vomiting, *n* (%)	0 (0.0)	0 (0.0)	1.00
PONV—day 2, *n* (%)	6 (9.0)	0 (0.0)	0.04
- Nausea not requiring treatment, *n* (%)	6 (9.0)	0 (0.0)	0.04
- Nausea requiring treatment, *n* (%)	0 (0.0)	0 (0.0)	1.00
- Vomiting, *n* (%)	0 (0.0)	0 (0.0)	1.00
Length of stay, mean ± SD, d	2.3 ± 0.5	2.3 ± 0.6	1.00

ADL: activities of daily living, IV: intravenous, PONV: postoperative nausea and vomiting, SD: standard deviation, VAS: visual analog scale.

## Data Availability

The raw data supporting the conclusions of this article will be made available by the authors on request.

## Abbreviations

The following abbreviations are used in this manuscript:

ERAS	Enhanced Recovery After Surgery
VAS	Visual Analog Scale
PONV	Postoperative Nausea and Vomiting
LOS	Length of Hospital Stay
CIN	Cervical Intraepithelial Neoplasia
CVA	Cerebrovascular Accident
HSIL	High-Grade Squamous Intraepithelial Lesion
IV	Intravenous
MI	Myocardial Infarction
SD	Standard Deviation
TIVA	Total Intravenous Anesthesia
ADL	Activities of Daily Living
